# Development of a 10K breeder-friendly SNP chip for faba bean

**DOI:** 10.3389/fpls.2026.1884871

**Published:** 2026-08-24

**Authors:** Hyeonah Shim, Hailin Zhang, Thomas Groß, Jörg Plieske, Heike Gnad, Stig Uggerhøj Andersen, Murukarthick Jayakodi

**Affiliations:** 1 Leibniz Institute of Plant Genetics and Crop Plant Research (IPK) Gatersleben, Seeland, Germany; 2 TraitGenetics Section, SGS Institut Fresenius GmbH, Seeland, Germany; 3 Department of Molecular Biology and Genetics, Aarhus University, Aarhus, Denmark; 4 Texas A&M AgriLife Research Center at Dallas, Texas A&M University System, Dallas, TX, United States

**Keywords:** faba bean, genetic diversity, high-throughput genotyping, legume genomics, SNP chip

## Abstract

**Introduction:**

Faba bean breeding and genomics have seen steady progress in recent years, supported by genome sequences and high-density genotyping platforms. These tools have been valuable for trait mapping, diversity assessment, and genomic research, but they have limited routine use in breeding programs due to their relatively high cost. Recent progress in establishing an optimized, cost-efficient genotyping-by-sequencing protocol tailored to the large and complex faba bean genome has created the foundation for a more accessible genotyping solution.

**Methods:**

Using this approach, we explored the genetic diversity of faba bean germplasm from various panels, providing a comprehensive representation of the crop’s genetic landscape. From this dataset, we identified and selected a high-quality set of informative SNP markers that are evenly distributed across the genome. Building on these resources, we designed a breeder-friendly 10K SNP chip.

**Results:**

The 10K SNP chip delivers high accuracy, broad genomic coverage, and affordability. The chip was validated across diverse germplasm panels, demonstrating strong clustering performance, high reproducibility, and applicability to breeding-relevant germplasm.

**Discussion:**

This platform offers a cost-effective alternative to higher-density arrays, enabling its integration into genomic selection, marker-assisted breeding, and diversity monitoring, ultimately supporting accelerated genetic gain and the delivery of improved varieties to farmers.

## 1 Introduction

Faba bean (*Vicia faba* L.) is one of the most important cool-season grain legumes worldwide, valued for its high protein content, adaptability to diverse environments, and role in sustainable cropping systems through biological nitrogen fixation (Schwenke et al., 1998; López-Bellido et al., 2006). Despite its agronomic importance, genetic improvement in faba bean has historically lagged behind that of many other crops, in part due to its exceptionally large and complex genome (~13 Gb) and the limited availability of efficient molecular tools for routine breeding (Johnston et al., 1999). Over the past decade, advances in sequencing technologies and genomic resources have accelerated the development of high-throughput genotyping approaches. Across legumes, single nucleotide polymorphism (SNP) arrays are available at a wide range of densities. For example, densities range from a few hundred markers, such as the 384-SNP Illumina GoldenGate assay developed for pea (Deulvot et al., 2010) to very high-density platforms such as the 355 K SoySNP array in soybean (Wang et al., 2016). Early efforts to develop high-throughput genotyping tools in faba bean led to the first SNP array based on cross-species resources, an initial array of 845 SNPs derived from *Medicago truncatula* sequences (Webb et al., 2016). Subsequently, higher-density platforms were developed, including the 60K Axiom SNP array (O’sullivan et al., 2019; Khazaei et al., 2021), 90K SPET marker set (Jayakodi et al., 2023), and the 130K targeted next-generation sequencing genotyping platform derived from transcriptome sequencing (Wang et al., 2021). These platforms have enabled genome-wide characterization of diversity, linkage mapping, and trait discovery, including large-scale diversity and GWAS analyses using existing faba bean SNP resources (Skovbjerg et al., 2023; Gutierrez et al., 2024). However, despite their high marker density and resolution, their routine deployment across breeding programs can still be constrained by cost, infrastructure requirements, or scalability for large sample numbers (Rasheed et al., 2017).

For practical breeding applications, a moderate-density, breeder-friendly SNP array can provide sufficient resolution for key applications such as genetic diversity analysis of breeding populations, parent selection, genomic selection, marker-assisted selection and quality control, while remaining cost-effective for large-scale routine use. The recent availability of a chromosome-level reference genome for faba bean (Jayakodi et al., 2023; Zhang et al., 2026), together with optimized genotyping-by-sequencing (GBS) protocols (Zhang et al., 2024) and targeted sequencing approaches such as SPET (Jayakodi et al., 2023), has provided a strong foundation for the development of a robust SNP resource. The integration of these complementary genomic tools enabled the identification of high-quality SNPs that are uniquely anchored to the reference genome and broadly distributed across chromosomes. Combining variant discovery from multiple platforms with a high-quality genome is therefore important for selecting markers with reliable genomic placement and suitable sequence context, supporting the development of a stable and reproducible assay for long-term breeding applications.

In this study, we report the development of a breeder-friendly 10K SNP chip for faba bean. Candidate markers were identified from multiple germplasm panels using complementary genotyping platforms and subjected to stringent filtering to ensure high quality, unique genomic placement, and even genome-wide distribution. The resulting marker set was evaluated across diverse panels representing elite breeding lines and broader germplasm collections to assess assay performance and its ability to resolve population structure.

## 2 Materials and methods

### 2.1 Discovery of high-quality SNPs

A diverse set of faba bean germplasm of 882 genotypes was used for marker discovery and selection, including the ProFaba, NORFAB and MAGIC panels (n = 672), which were genotyped using the previously developed SPET markers from Jayakodi et al. (2023), and a panel of elite breeding lines (n = 210) genotyped using genotyping-by-sequencing (GBS) following the protocol of Zhang et al. (2024) and whole genome resequencing (Zhang et al., 2026). Variants were called within each platform and subjected to stringent quality filtering to obtain a robust pool of candidate loci for array design. Only biallelic SNPs were retained and filtered for minor allele frequency ≥ 5% and heterozygosity < 5% across samples. To ensure probe reliability, candidate loci were excluded if ≥ 2 SNPs and/or indels were present within a 100 bp window surrounding the target site. For each retained SNP, 75 bp flanking sequences on both sides were extracted and aligned to the reference genome (Zhang et al., 2026), and only markers showing a single unique genomic location were kept.

Marker filtering and selection were performed separately for the SPET-derived and GBS-derived datasets prior to integration into the final chip design. To achieve genome-wide coverage and minimize redundancy, filtered variants were evaluated in fixed 200 kb physical windows across each chromosome separately for the SPET-derived and GBS-derived datasets. Within each window, markers were prioritized based on allele frequency, call quality, and suitability for probe design, and linkage disequilibrium (LD) pruning was applied to remove highly correlated loci and retain representative markers. Candidate sets from SPET and GBS panels were then merged. Platform-specific markers were retained to improve genome coverage and polymorphism across breeding materials. From this combined pool, a final set of evenly distributed, high-quality SNPs was selected to form the basis of the faba bean SNP chip.

### 2.2 Assay validation

Genotyping was performed using the 10K faba bean Infinium XT array on a 96-sample BeadChip format. A total of 366 samples were processed across four plates, including elite breeding lines (n = 190), ProFaba panel lines (n = 88), and winter panel lines (n = 88). High molecular weight DNA of approx. 20 ng/µl of each of the lines described above was analyzed according to the Illumina Infinium XT protocol (reference guides available at https://support.illumina.com). The chips were scanned on the Illumina iScan and the image files were analyzed with the Illumina^®^ Genome Studio 2.0.5 (GS) software. Cluster calling of each marker was manually optimized for construction of an optimized cluster file for genotyping. The final report files for the generation of the genotype table were extracted from the GS software using the respective manifest for the 10K faba bean chip according to the source sequence.

To assess genotyping consistency across platforms, genotype concordance between the 10K SNP chip and SPET and GBS datasets was evaluated for 212 overlapping accessions. Shared SNP loci between datasets were identified based on common genomic coordinates, and allele orientations were aligned prior to comparison. Genotype concordance was assessed using bcftools gtcheck (v1.15.1) and by calculating the proportion of matching genotype calls across shared markers and overlapping accessions.

### 2.3 Genetic diversity

Genetic diversity was examined using principal component analysis (PCA) across three diversity panels: the Göttingen winter panel (n=88), elite lines (n=190), and the ProFaba panel (n=88). The SNP markers generated from the 10K chip were filtered prior to analysis, and markers with excessive missing data or low minor allele frequency were excluded. The PCA was performed using --pca function in PLINK2 (Chang et al., 2015), computing eigenvalues and eigenvectors from the genotype covariance matrix. The first principal components were used to visualize genetic structure among panels. The PCA plots were generated in R using the ggplot2 package.

## 3 Results

### 3.1 Genome-wide SNP discovery

Application of a minor allele frequency threshold (MAF ≥ 5%) yielded 239,850 candidate SNPs from the SPET panels and 16,545 from the GBS panel that were anchored to chromosomes. Subsequent filtering for probe suitability and variant quality progressively reduced the marker set. After enforcing the requirement of ≤2 SNPs and/or indels within a 100 bp window around the target site and retaining only biallelic loci, 55,437 SPET SNPs and 11,306 GBS SNPs remained. Requiring unique mapping of 75 bp flanking sequence to a single genomic position further reduced the set to 49,194 and 7,054 SNPs, respectively. Filtering for excess heterozygosity (< 5%) resulted in 32,761 SPET and 7,012 GBS high-confidence candidate markers.

**Figure 1:**
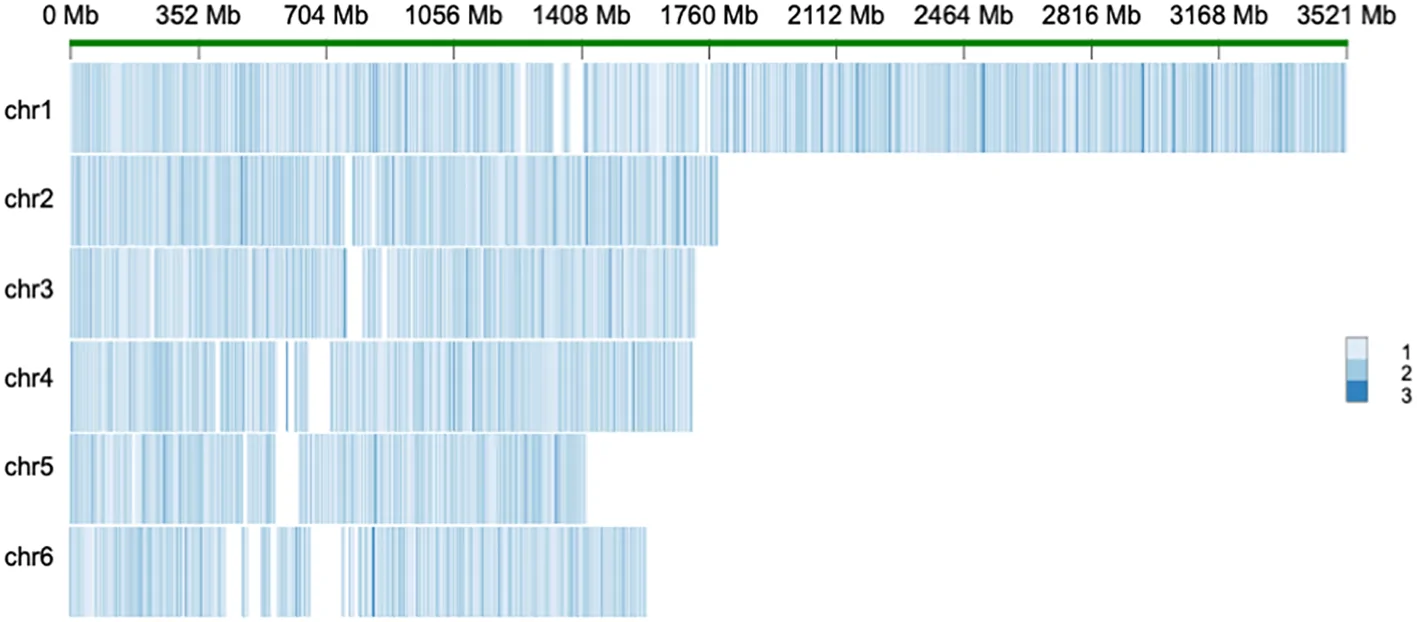
Genome-wide distribution of single nucleotide polymorphisms (SNPs). Physical distribution of detected SNPs across chromosomes 1 to 6. The lightest bars represent 1 SNP and darkest blue bars represent three SNPs within the genomic window.

To ensure genome-wide representation and reduce redundancy, markers were evaluated in 200 kb physical windows across chromosomes. This step resulted in 12,289 SPET-derived and 5,807 GBS-derived SNPs distributed across the genome. After merging both datasets and collapsing overlapping loci, a combined pool of 18,063 candidate SNPs was obtained. From this set, markers were further selected to achieve balanced genome coverage, resulting in a final selection of 12,153 SNPs for the breeder-friendly chip, comprising 9,151 SPET-derived and 3,028 GBS-derived markers. The markers were evenly distributed along each of the chromosomes (**Figure 1**).

### 3.2 Marker selection for 10K SNP chip

The initial marker set showed a balanced distribution across all six faba bean chromosomes, ensuring genome-wide coverage suitable for breeding and diversity analyses. In total, 7,791 SNPs were retained following initial array design and clustering, of which 1,741 overlapped with loci identified in the GBS dataset. The number of SNPs per chromosome ranged from 915 to 2,424, with 2,424 SNPs on chromosome 1 (3.52 Gb), 1,246 on chromosome 2 (1.78 Gb), 1,116 on chromosome 3 (1.72 Gb), 1,121 on chromosome 4 (1.71 Gb), 915 on chromosome 5 (1.42 Gb), and 969 on chromosome 6 (1.59 Gb). This distribution broadly reflected differences in chromosome size and marker availability, with window-based marker prioritization used to maintain even spacing across the genome.

**Figure 2:**
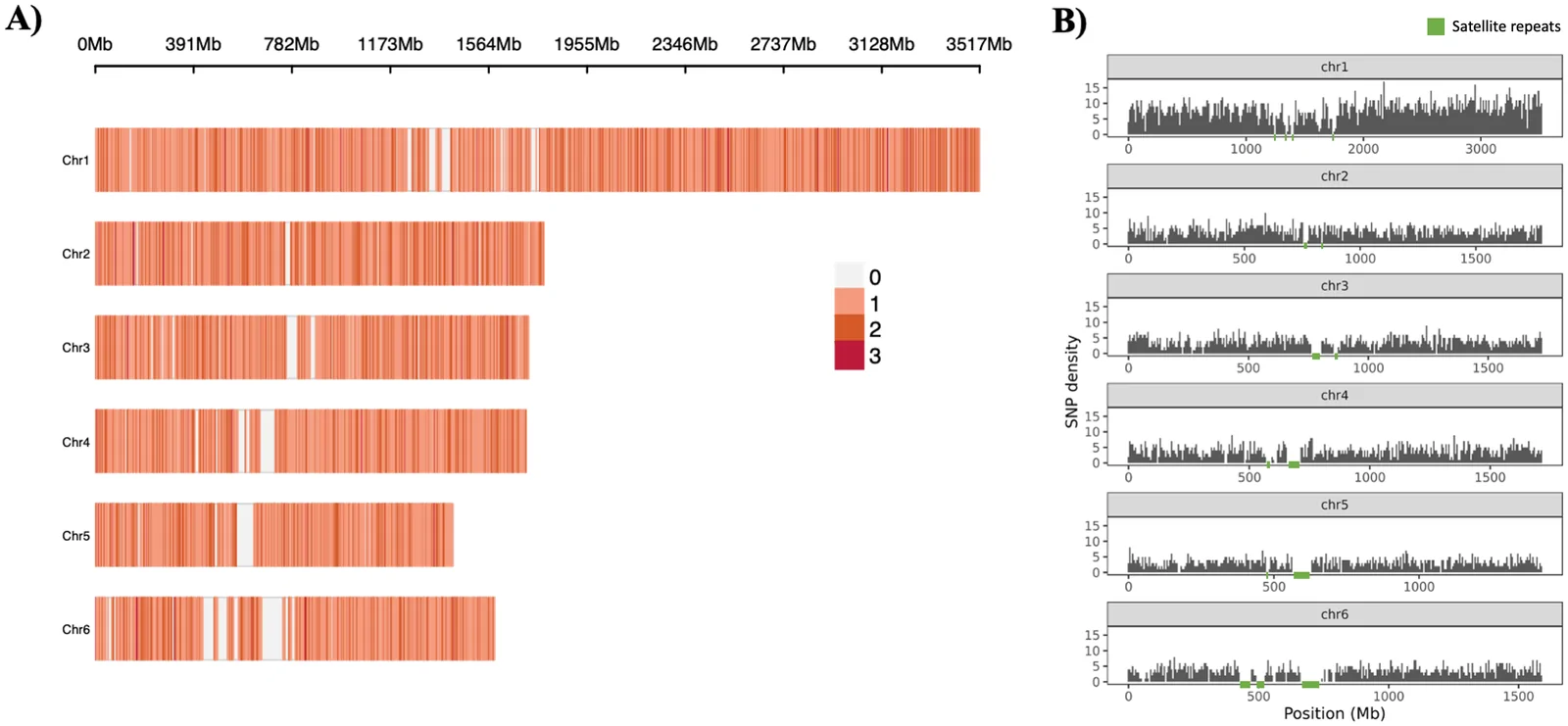
Genome-wide distribution of selected markers for the SNP chip. Physical distribution of SNPs included in the final breeder-friendly chip across chromosomes 1 to 6. **(A)** Distribution of SNPs throughout each chromosome. Color scale indicates 0 (white) to 3 (dark red) markers per window. **(B)** Each vertical bar represents the density of selected markers within genomic windows along the chromosome. Green rectangles along the x-axis indicate regions of satellite repeat clusters.

After applying post-genotyping quality filters (<10% missing data and <10% heterozygosity), 6,246 high-confidence SNPs remained, retaining representation across all chromosomes (**Figure 2**). This post-filtered final marker set distribution demonstrated consistent genome-wide coverage without major gaps, indicating that the selection strategy successfully achieved an evenly distributed and robust marker set for downstream breeding and genetic analyses.

### 3.3 Assessing the SNP chip

Application of the 10K faba bean SNP chip to the panels produced high genotype call rates across the three breeding panels (**Table 1**). The mean genotype call rate was highest in the elite panel (94.75%), followed by the Göttingen winter panel (92,32%) and the ProFaba panel (90.49%). These results indicate that the marker set generated genotype data with consistently high completeness across genetically diverse materials.

**Table 1:** Faba bean germplasm panels and 10K SNP chip genotype call rates.

Panel	No. of samples	Genotype call rate (%)
Elite	188	94.75
ProFaba	90	90.49
Winter	90	92.32

**Figure 3:**
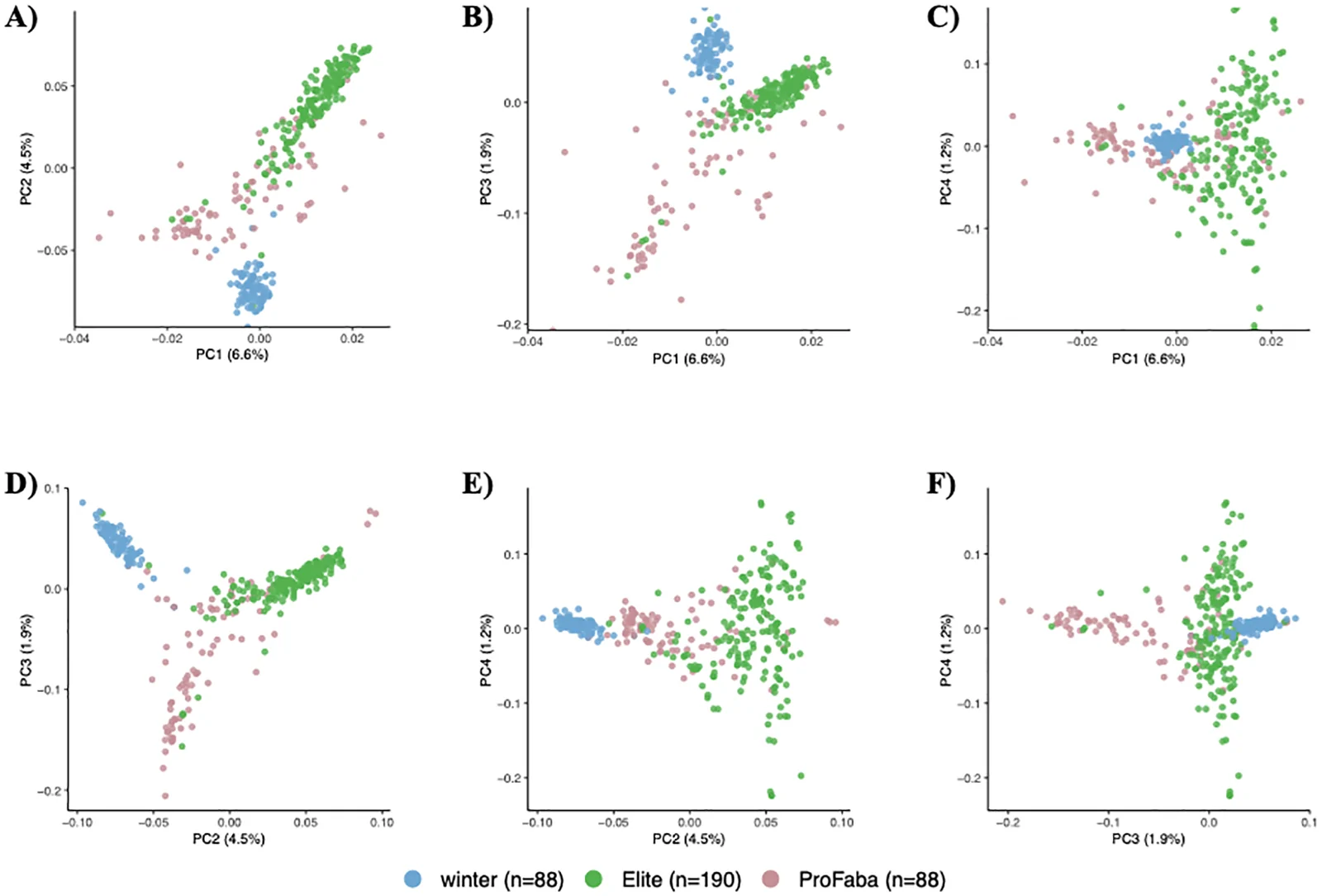
Principal component analysis of the faba bean diversity panels genotyped with the 10K SNP chip. PCA was conducted using the filtered marker set for 366 accessions comprising elite lines (green; n = 190), the Göttingen winter panel (blue; n = 88), and ProFaba lines (brown; n = 88). All pairwise combinations among the first four principal components are shown: **(A)** PC1–PC2, **(B)** PC1–PC3, **(C)** PC1–PC4, **(D)** PC2–PC3, **(E)** PC2–PC4, and **(F)** PC3–PC4. PC1, PC2, PC3, and PC4 explained 6.6%, 4.5%, 1.9%, and 1.2% of the total genetic variance, respectively. The plots illustrate broad patterns of genetic similarity and differentiation among the three panels.

Principal component analysis (PCA) based on the filtered marker set was subsequently used to visualize broad patterns of genetic similarity and differentiation among the three panels (**Figure 3**). All six pairwise combinations among the first four principal components were examined. PC1, PC2, PC3, and PC4 explained 6.6%, 4.5%, 1.9%, and 1.2% of the total genetic variance, respectively. The clearest differentiation was observed in the PC1–PC2 comparison, in which the Göttingen winter accessions formed a compact cluster separated from most elite and ProFaba materials. The elite panel showed a broader distribution, whereas the ProFaba accessions were more dispersed and partially overlapped with the elite panel. Comparisons involving PC3 and PC4 revealed additional variation within and among the panels. Together, the high genotype call rates and the broad differentiation observed in the PCA demonstrate that the 10K SNP chip provides sufficient resolution for diversity analysis and germplasm characterization and offers a practical tool for applications such as parent selection, population structure analysis, and genomic-assisted breeding (Webb et al., 2016; Skovbjerg et al., 2023).

Genotype consistency between the 10K SNP chip and other genotyping platforms was assessed using 212 overlapping accessions shared with GBS and SPET datasets. A total of 528 and 2,173 shared SNP markers were retained for the GBS versus 10K SNP chip and SPET versus 10K SNP chip comparisons, respectively. Mean genotype concordance across overlapping samples was 74.5% for the GBS comparison and 71.2% for the SPET comparison, supporting the overall consistency of genotype calls across platforms.

## 4 Discussion

Multiple marker sets and genotyping platforms are available for faba bean, but these resources are often not complementary, and their routine use in breeding programs remains constrained by cost and limited flexibility. The 10K SNP chip developed in this study was designed as a cost-effective genome-wide platform with balanced genome coverage to support breeding applications. The SNPs included in the array were selected as genome-wide markers based on genomic distribution, sequence quality, allele frequency, unique placement in the reference genome, and suitability for array design, rather than on known associations with specific traits. Therefore, the primary practical applications of the chip include the assessment of genetic relatedness and population structure in breeding populations, quality control of breeding materials, parent selection, and the generation of genome-wide marker data for genomic prediction.

Marker selection was facilitated by the availability of a reference genome (Zhang et al., 2026) and incorporated SNPs derived from multiple datasets to capture a range of genetic diversity. The markers were validated using both diversity panels and breeding populations, ensuring robust performance across relevant germplasm. By combining curated high-quality SNPs with genome-anchored positions, the chip provides improved genome representation and positional confidence compared with earlier faba bean marker resources developed before the availability of chromosome-level genome assemblies, including consensus SNP maps, the Vfaba_v2 Axiom SNP array, targeted genotyping platforms, and GBS-based resources (Webb et al., 2016; O’sullivan et al., 2019; Khazaei et al., 2021; Wang et al., 2021; Zhang et al., 2024). Despite some overlap among breeding materials, individuals from each panel clustered predominantly with their respective groups, indicating that the marker set captures meaningful genome-wide variation and population structure, notably the winter genotypes. The genotype concordance observed among overlapping lines across platforms provides additional support for the reliability of the 10K chip, while also highlighting expected differences among genotyping approaches. The observed concordance of 74.5% and 71.2% suggests moderate to substantial agreement between genotyping approaches. The remaining discordance likely reflects platform-specific differences in marker ascertainment, sequencing depth and missingness, read alignment, genotype-calling procedures, and, where applicable, differences in the exact DNA source or biological material used for each accession.

The genome-wide and well-anchored marker set can support downstream genetic analyses, including QTL mapping, and genomic prediction. Following trait association and validation in relevant breeding populations, informative SNPs identified could be converted into flexible assays such as KASP markers for targeted use in marker-assisted selection. Similar faba bean SNP resources have supported trait mapping and marker discovery for agronomic and seed-quality traits, including vicine-convicine content (Gutierrez et al., 2006; Khazaei et al., 2015; Jayakodi et al., 2023), tannin levels (Gutierrez et al., 2007), hilum color (Khazaei et al., 2015; Zhang et al., 2024), flowering time and seed quality traits (Karaköy et al., 2024; Ohm et al., 2024). The marker set generated in this study is evenly distributed across all chromosomes with minimal clustering and limited genomic gaps, achieved through stringent filtering criteria including minimum allele frequency thresholds, low heterozygosity, and selection of markers in single-copy regions. This design supports consistent genome coverage and enhances the reliability of downstream analyses.

## Data Availability

The genotype data generated and analyzed in this study are publicly available in Figshare at https://doi.org/10.6084/m9.figshare.33253590.

## References

[B1] Chang C. C. Chow C. C. Tellier L. C. Vattikuti S. Purcell S. M. Lee J. L. (2015). Second-generation PLINK: rising to the challenge of larger and richer datasets. GigaScience 4, s13742-015-0047-8. doi: 10.1186/s13742-015-0047-8 25722852 PMC4342193

[B2] Deulvot C. Charrel H. Marty A. Jacquin F. Donnadieu C. Lejeune-Hénaut I. . (2010). Highly-multiplexed SNP genotyping for genetic mapping and germplasm diversity studies in pea. BMC Genomics 11, 468. doi: 10.1186/1471-2164-11-468 20701750 PMC3091664

[B3] Gutierrez N. Avila C. Duc G. Marget P. Suso M. Moreno M. T. . (2006). CAPs markers to assist selection for low vicine and convicine contents in faba bean (Vicia faba L.). Theor. Appl. Genet. 114, 59–66. doi: 10.1007/s00122-006-0410-3 17013617

[B4] Gutierrez N. Ávila C. M. Rodríguez-Suárez C. Moreno M. Torres A. M. (2007). Development of SCAR markers linked to a gene controlling absence of tannins in faba bean. Mol. Breed. 19, 305–314. doi: 10.1007/s11032-006-9063-9 30311153

[B5] Gutierrez N. Pégard M. Solis I. Sokolovic D. Lloyd D. Howarth C. . (2024). Genome-wide association study for yield-related traits in faba bean (Vicia faba L.). Front. Plant Sci. 15, 1328690. doi: 10.3389/fpls.2024.1328690 38545396 PMC10965552

[B6] Jayakodi M. Golicz A. A. Kreplak J. Fechete L. I. Angra D. Bednář P. . (2023). The giant diploid faba genome unlocks variation in a global protein crop. Nature 615, 652–659. doi: 10.1038/s41586-023-05791-5 36890232 PMC10033403

[B7] Johnston J. S. Bennett M. D. Rayburn A. L. Galbraith D. W. Price H. J. (1999). Reference standards for determination of DNA content of plant nuclei. Am. J. Bot. 86, 609–613. doi: 10.2307/2656569 10330063

[B8] Karaköy T. Toklu F. Karagöl E. T. Uncuer D. Çilesiz Y. Ali A. . (2024). Genome-wide association studies revealed DArTseq loci associated with agronomic traits in Turkish faba bean germplasm. Genet. Resour. Crop Evol. 71, 181–198. doi: 10.1007/s10722-023-01615-7

[B9] Khazaei H. O'Sullivan D. M. Stoddard F. L. Adhikari K. N. Paull J. G. Schulman A. H. . (2021). Recent advances in faba bean genetic and genomic tools for crop improvement. Legume. Sci. 3, e75. doi: 10.1002/leg3.75 34977588 PMC8700193

[B10] Khazaei H. O'Sullivan D. M. Jones H. Pitts N. Sillanpää M. J. Pärssinen P. . (2015). Flanking SNP markers for vicine–convicine concentration in faba bean (Vicia faba L.). Mol. Breed. 35, 38. doi: 10.1007/s11032-015-0214-8 30311153

[B11] López-Bellido L. López-Bellido R. J. Redondo R. Benítez J. (2006). Faba bean nitrogen fixation in a wheat-based rotation under rainfed Mediterranean conditions: Effect of tillage system. Field Crops Res. 98, 253–260. doi: 10.1016/j.fcr.2006.03.001 38826717

[B12] O’sullivan D. Angra D. Harvie T. Tagkouli V. Warsame A. (2019). 123. A genetic toolbox for Vicia faba improvement. For. People Planet., 157.

[B13] Ohm H. Åstrand J. Ceplitis A. Bengtsson D. Hammenhag C. Chawade A. . (2024). Novel SNP markers for flowering and seed quality traits in faba bean (Vicia faba L.): characterization and GWAS of a diversity panel. Front. Plant Sci. 15, 1348014. doi: 10.3389/fpls.2024.1348014 38510437 PMC10950902

[B14] Rasheed A. Hao Y. Xia X. Khan A. Xu Y. Varshney R. K. . (2017). Crop breeding chips and genotyping platforms: progress, challenges, and perspectives. Mol. Plant 10, 1047–1064. doi: 10.1016/j.molp.2017.06.008 28669791

[B15] Schwenke G. Peoples M. Turner G. Herridge D. (1998). Does nitrogen fixation of commercial, dryland chickpea and faba bean crops in north-west New South Wales maintain or enhance soil nitrogen? Aust. J. Exp. Agric. 38, 61–70. doi: 10.1071/ea97078 38477348

[B16] Skovbjerg C. K. Angra D. Robertson-Shersby-Harvie T. Kreplak J. Keeble-Gagnère G. Kaur S. . (2023). Genetic analysis of global faba bean diversity, agronomic traits and selection signatures. Theor. Appl. Genet. 136, 114. doi: 10.1007/s00122-023-04360-8 37074596 PMC10115707

[B17] Wang J. Chu S. Zhang H. Zhu Y. Cheng H. Yu D. (2016). Development and application of a novel genome-wide SNP array reveals domestication history in soybean. Sci. Rep. 6, 20728. doi: 10.1038/srep20728 26856884 PMC4746597

[B18] Wang C. Liu R. Liu Y. Hou W. Wang X. Miao Y. . (2021). Development and application of the Faba_bean_130K targeted next-generation sequencing SNP genotyping platform based on transcriptome sequencing. Theor. Appl. Genet. 134, 3195–3207. doi: 10.1007/s00122-021-03885-0 34117907

[B19] Webb A. Cottage A. Wood T. Khamassi K. Hobbs D. Gostkiewicz K. . (2016). A SNP‐based consensus genetic map for synteny‐based trait targeting in faba bean (Vicia faba L.). Plant Biotechnol. J. 14, 177–185. doi: 10.1111/pbi.12371 25865502 PMC4973813

[B20] Zhang H. Fechete L. I. Himmelbach A. Poehlein A. Lohwasser U. Börner A. . (2024). Optimization of genotyping‐by‐sequencing (GBS) for germplasm fingerprinting and trait mapping in faba bean. Legume. Sci. 6, e254. doi: 10.1002/leg3.254 41531421

[B21] Zhang H. Windhorst A. Bornhofen E. Tulpova Z. Novak P. Macas J. . (2026). Allelic variation at a single locus distinguishes spring and winter faba beans. Nat. Genet. 58, 655–663. doi: 10.1038/s41588-026-02524-y 41807799 PMC12987728

